# Effects and mechanism of Aβ_1−42_ on EV-A71 replication

**DOI:** 10.1186/s12985-022-01882-3

**Published:** 2022-09-20

**Authors:** Ming Zhong, Huiqiang Wang, Haiyan Yan, Shuo Wu, Kun Wang, Lu Yang, Boming Cui, Mengyuan Wu, Yuhuan Li

**Affiliations:** 1 CAMS Key Laboratory of Antiviral Drug Research, Institute of Medicinal Biotechnology, Chinese Academy of Medical Sciences and Peking Union Medical College, 1 Tiantan xili, Beijing, 100050 China; 2 Beijing Key Laboratory of Antimicrobial Agents, Institute of Medicinal Biotechnology, Chinese Academy of Medical Sciences and Peking Union Medical College, Beijing, 100050 China

**Keywords:** β-Amyloid protein, Enterovirus A 71, Capsid protein VP1, Scavenger receptor class B member 2

## Abstract

**Background:**

β-Amyloid (Aβ) protein is a pivotal pathogenetic factor in Alzheimer’s disease (AD). However, increasing evidence suggests that the brain has to continuously produce excessive Aβ to efficaciously prevent pathogenic micro-organism infections, which induces and accelerates the disease process of AD. Meanwhile, Aβ exhibits activity against herpes simplex virus type 1 (HSV-1) and influenza A virus (IAV) replication, but not against other neurotropic viruses. Enterovirus A71 (EV-A71) is the most important neurotropic enterovirus in the post-polio era. Given the limitation of existing research on the relationship between Aβ and other virus infections, this study aimed to investigate the potent activity of Aβ on EV-A71 infection and extended the potential function of Aβ in other unenveloped viruses may be linked to Alzheimer's disease or infectious neurological diseases.

**Methods:**

Aβ peptides 1–42 are a major pathological factor of senile plaques in Alzheimer’s disease (AD). Thus, we utilized Aβ_1–42_ as a test subject to perform our study. The production of monomer Aβ_1–42_ and their high-molecular oligomer accumulations in neural cells were detected by immunofluorescence assay, ELISA, or Western blot assay. The inhibitory activity of Aβ_1–42_ peptides against EV-A71 in vitro was detected by Western blot analysis or qRT-PCR. The mechanism of Aβ_1–42_ against EV-A71 replication was analyzed by time-of-addition assay, attachment inhibition assay, pre-attachment inhibition analysis, viral-penetration inhibition assay, TEM analysis of virus agglutination, and pull-down assay.

**Results:**

We found that EV-A71 infection induced Aβ production and accumulation in SH-SY5Y cells. We also revealed for the first time that Aβ_1–42_ efficiently inhibited the RNA level of EV-A71 VP1, and the protein levels of VP1, VP2, and nonstructural protein 3AB in SH-SY5Y, Vero, and human rhabdomyosarcoma (RD) cells. Mechanistically, we demonstrated that Aβ_1–42_ primarily targeted the early stage of EV-A71 entry to inhibit virus replication by binding virus capsid protein VP1 or scavenger receptor class B member 2. Moreover, Aβ_1–42_ formed non-enveloped EV-A71 particle aggregates within a certain period and bound to the capsid protein VP1, which partially caused Aβ_1–42_ to prevent viruses from infecting cells.

**Conclusions:**

Our findings unveiled that Aβ_1–42_ effectively inhibited nonenveloped EV-A71 by targeting the early phase of an EV-A71 life cycle, thereby extending the potential function of Aβ in other non-envelope viruses linked to infectious neurological diseases.

**Supplementary Information:**

The online version contains supplementary material available at 10.1186/s12985-022-01882-3.

## Background

β-Amyloid (Aβ) is identified as an essential pathological factor involved in Alzheimer's disease (AD) [1, 2]. Nevertheless, a deeper understanding of the relationship between AD and pathogenic microbial infections is prompting further exploration of the physiological function of Aβ. Aβ, a novel defined antimicrobial peptide (AMP), also reportedly exhibits antiviral action against herpes simplex virus type 1 (HSV-1) and influenza A virus (IAV) [3–6]. The oligomerization of Aβ peptide prevents bacteria from binding to bacterial-surface carbohydrates, which is also the partial mechanism of Aβ in exerting its anti-herpes virus effect [4, 5, 7]. Meanwhile, Bourgade et al*.* suggested that Aβ may be associated with another favorable antiviral mechanism in addition to the carbohydrate-binding-mediated antiviral pathway [3].

Enterovirus A 71 (EV-A71), a non-enveloped virus with a positive-sense ssRNA genome enclosed within an icosahedron capsid shell, comprises 60 copies of four capsid proteins, VP1, VP2, VP3, and VP4 [8]. For non-enveloped viruses, the binding of receptors is a critical event in the course of infection. EV-A71 utilizes cell-surface glycoproteins as receptors to infect host cells [9, 10]. Scavenger receptor class B member 2 (SCARB2), a type III multichannel membrane glycoprotein, is the main receptor for EV-A71 on target cells [11–13]. SCARB2 is pivotal in EV-A71’s attachment and uncoating, facilitating efficient EV-A71 infection [14].

Besides causing hand, foot and mouth disease (HFMD), EV-A71 also infects the CNS and induces neurology-related severe diseases [15]. The relationship between EV-A71 and Aβ has not been reported. In the present study, we demonstrated for the first time that EV-A71 infection induced Aβ_1–42_ production and accumulation and elucidated the mechanism by which Aβ_1–42_ inhibited EV-A71 replication. Our results revealed that Aβ presented antiviral activity against EV-A71 infection, paving the way for further research on the potential role of Aβ peptides in other brain-infecting viruses.

## Methods

### Oligomeric synthetic peptide preparation and compounds

Aβ peptides 1–42 are a major pathological factor of senile plaques in AD [16, 17]. Accordingly, we utilized Aβ_1–42_ as a test subject to perform our study.

Synthetic Aβ_1–42_ peptides were obtained from Anaspec (Fremont, CA. USA). Dried peptides were solubilized in 150 mM NaCl prior to experimentation. Aβ_1–42_ was used at 30 µg/mL concentration for the viral life-cycle experiment and 20 µg/mL for the antiviral activity test or pull-down experiment.

Pirodavir was purchased from Biochempartner (Shanghai, China), and NH_4_Cl was bought from MedChemExpress (USA). Stock solutions of pirodavir (10 mM) and NH_4_Cl (100 mM) were prepared in dimethyl sulfoxide (Sigma–Aldrich, Carlsbad, CA, USA), respectively. RBV stock solutions (2 mg/mL) were dissolved in a cell culture medium.

### Viral strains and cell lines

Human rhabdomyosarcoma (RD) cells and SH-SY5Y cells were bought from the Cellular Cultivation Center of Peking Union Medical College (PUMC) or Chinese Academy of Sciences (CAS) and cultivated in Dulbecco’s adjusted Eagle intermediary comprising 10% fetal bovine serum (FBS; Gibco, USA) and 1% penicillin–streptomycin (Invitrogen, Carlsbad, CA, USA). Vero cells were acquired from the American Type Culture Collection (ATCC), and cultured in modified Eagle’s medium (Invitrogen, Carlsbad, CA, USA) supplemented with 10% inactivated FBS (Gibco, Grand Island, NY, USA) and 1% penicillin–streptomycin.

EV-A71 strain H (VR-1432) and Coxsackievirus B3 strain Nancy (VR-30) were purchased from ATCC. CVA16 strain (shzh05-1/GD/CHN/2005) was offered by Dr. Jianwei Wang, Institute of Pathogen Biology, CAS, and PUMC. Entire viral strains were passaged in Vero cells.

### Quantification of Aβ_1–42_ by ELISA

A human Aβ (1–42 aa) Quantikine ELISA Kit (R&D Systems, DAB142) was used to identify human Aβ (aa1–42) in cellular cultivation supernatant under the condition of EV-A71 infection. The experiment was performed per the manufacturer’s instructions.

### Cytotoxicity assay

The cytotoxic effects of Aβ_1–42_ peptides on SH-SY5Y, Vero, and RD cells were evaluated with a Cell-Counting Kit (CCK) (TransGen Biotech, Beijing, China). In a typical procedure, cells were cultured in 96-well plates and indicated concentrations of Aβ_1–42_ peptides were added for 48 h. Then, 10 µL of CCK solution was added to each cell well. The absorbance of the plates was detected at 450 nm on an Enspire system (PerkinElmer, Waltham, MA, USA) after incubating at 37 °C for 30 min.

### Western blot (WB) assay

Total proteins were lysed with an M-PER mammal-protein abstraction reagent containing a halt protease suppressor cocktail (Thermo Fisher Scientific, USA). An equal amount of cell lysate was used in a 10%–12% (w/v) SDS-PAGE gels, electro-transferred onto PVDF membranes (Millipore, USA), and blocked with 5% (w/v) milk at room temperature for 2 h. The films were incubated with the first antibody against EV-A71 VP1 (Abnova, Taipei, China), EV-A71 VP2 (GeneTex, California, USA), EV-A71 3AB (GeneTex, California, USA), SCARB2 (Abcam, UK), Aβ_1–42_ (CST, USA), and β-actin (CST, USA) overnight at 4 °C. Finally, an ECL identification kit (GE Healthcare Life Sciences, USA) was used with an appropriate second antibody (CST, USA) for 1 h at room temperature [18]. Software “Gel-Pro analyzer” was used to analysis of the optical density ratio of the bands.

To detect of Aβ1–42 monomer (4 kDa), the suspensions were mixed with 1 × loading buffer (containing DTT) and boiled for 10 min. We used 20% Tris–Tricine–SDS-PAGE gels prepared per the manufacturer’s instructions (Solarbio, China). The gels were transferred onto PVDF membranes for 1 h under the condition of 200 mA by using a transfer buffer containing 20% methanol, 190 mM glycine, and 25 mM Tris. The membranes were blocked with 5% (w/v) milk at room temperature for 2 h. The films were incubated with the first antibody against Aβ_1–42_ (CST, USA) overnight at 4 °C. Finally, an ECL identification kit (GE Healthcare Life Sciences, USA) was used with an appropriate second antibody (CST, USA) for 1 h at room temperature [18].

### Immunofluorescence assay and confocal microscopy

SH-SY5Y and Vero cells were infected with EV-A71 (MOI = 1) for 8 h. Cells were fixed with 4% polyoxymethylene for 30 min before the cultivation in 0.1% Triton X-100 for another 20 min. Cells were then blocked and cultivated with an antibody against VP1 and Aβ_1–42_. After washing in TBS three times, VP1 protein was visualized using an Alexa Fluor 488-conjugated secondary antibody (Invitrogen), whereas Aβ_1–42_ protein was visualized with Alexa Fluor 594. Cell nuclei were dyed with DAPI (Beyotime, PRC). Images were captured with an Olympus TH4-200 microscope or PE UltraVIEW VOX.

### Real-time reverse transcription-PCR (qRT-PCR)

Overall, RNA was extracted with RNeasy Mini Kit (Qian, USA). The level of EV-A71 VP1 RNA was quantified with One-Step qRT-PCR by using primers VP1-forward (5′-GATATCCCACATTCGGTGA-3′) and VP1-reverse (5′-TAGGACACGCTCCATACTCAAG-3′). The level of CVA16 VP1 RNA was identified using primers VP1-forward (5′-GTTATCCCACCTTCGGAGA-3′) and VP1-reverse (5′-TCGGGCATTGACCATAATCTAG-3′). The level of CVB3 VP1 RNA was amplified with forwarding primers VP1 (5′-TGCTCCGCAGTTAGGATTAGC-3′) and reverse VP1 (5′-ACATGGTGCGAAGAGTCTATTGAG-3′). GAPDH mRNA and β-actin mRNA served as an internal control to standardize the examined mRNAs by using primers GAPDH-forward (5′-GAAGGTGAAGGTCGGAGTC-3′). GAPDH-reverse (5′-GAAGATGGTGATGGGATTTC-3′). The relative fold change of the detected RNA specimens was analyzed by the comparative 2^−ΔΔCT^ method [19].

### Time-of-addition assay

The virus-replication steps targeted by Aβ_1–42_ were mapped by identifying the role of sequential supplementation of Aβ_1–42_ on EV-A71 VP1-level variation. In short, SH-SY5Y cells were subjected to EV-A71 infection (MOI = 10), and Aβ_1–42_ (30 μg/mL) was supplemented at the time of infection or at a different time post-infection. Entire cells were collected at 8 h post-infection, and VP1 expression was detected by WB assay.

### Attachment-inhibition assay

Cell plates were stored at 4 °C for 60 min before starting the assay. SH-SY5Y, Vero, or RD cells were incubated with EV-A71 (MOI = 2.5) and Aβ_1–42_ (30 µg/mL) at 4 °C for 60 min. Afterwards, cells were washed in cold PBS (pH = 7.4) three times and subjected to a qRT-PCR assay.

### Pre-attachment inhibition analysis

The pre-attachment inhibition analysis was designed as two experimental methods to test the various suppression characteristics of Aβ_1–42_.

For the pre-attachment inhibition assay, EV-A71 virus (MOI = 2.5) was mixed with Aβ_1–42_ (30 µg/mL) peptides at 4 °C for 1 h and attached to SH-SY5Y or Vero cells in cold 12-well plates at 4 °C for another 1 h. Total cellular RNA was extracted after three washes with cold PBS (pH = 7.4) and analyzed by qRT-PCR.

For the pre attachment inhibition assay of Aβ_1–42_ peptides, 20 µg/mL Aβ_1–42_ was incubated with precooled cell plates at 4 °C for 60 min and then washed in cold PBS three times. The processed cells were used in the subsequent pre attachment inhibition assays of the virus.

### Virus-penetration inhibition assay

Cells were cultivated with EV-A71 (MOI = 2.5) at 4 °C for 60 min to enable viral attachments, followed by cleaning in cold PBS three times to realize the removal of nonbound viruses. They were subsequently cultivated at 37 °C for 60 min to internalize viruses, and uncoating occurred eventually. After washing the unbound viruses three times, cells were lysed, and the RNA content of EV-A71 was measured by qRT-PCR analysis.

### Purification of EV-A71 virions

EV-A71 was propagated in Vero cells at 37 °C for 3 days. The cells were freeze-thawed in three cycles and then centrifuged at 3000 g for 30 min to remove cell debris. EV-A71 was initially concentrated using a 100 kDa centrifugal concentrator (Millipore, Billerica, MA, USA) at 3000 g for 30 min. Then, the virus was sedimented through a 20% density sucrose layer at 4 °C, 14,000 g for 3 h with a Beckman SW41 Ti rotor. Virus fractions at the bottom were harvested and gently resuspended in PBS (pH = 7.4). The purified EV-A71 stock was supplied for transmission electron microscopy (TEM) and pull-down assays [5, 6].

### TEM analysis of virus agglutination

The purified EV-A71 stock was cultivated together with Aβ_1–42_ peptides in PBS (pH = 7.4) at 4 °C for 1 h, absorbed into formvar carbon-coated copper grids for 60 s, and then stained with 1% (w/v) phosphotungstic acid (pH = 6.8) for another 60 s. The grids were desiccated in air atmosphere and imaged with a Tecnai12 TEM (FEI, Eindhoven, Netherlands) with a CCD camera (EMSIS MRADA g3, Germany).

### Pull-down assay

For EV-A71 VP1 pull-down assays, 20 µg/mL Aβ_1–42_ was incubated with protein A/G magnetic beads at 4 °C overnight before adding IgG or purified EV-A71 after cleaning in cold PBS three times. Subsequently, the mixture was cultivated at 4 °C for 2 h. After cleaning in cold PBS three times, the bound virions were detected by WB assay with VP1 antibody.

For the SCARB2 pull-down assays, 6 µg of SCARB2-Myc plasmid or pcDNA 3.1 + vector was transfected into Vero cells and lysed with protein lysate containing protein phosphatase and protease inhibitor at 24 h post-transfection. Subsequently, the lysis buffer supernatant was mixed with Aβ_1–42_ immobilized magnetic protein A/G magnetic beads at 4 °C for 2 h. The bounded beads were suspended with a 1 × sample loading buffer and boiled for 10 min. The binding of SCARB2 was detected by WB with anti-SCARB2 antibody.

### Statistical analysis

The two groups were contrasted by Student’s T-test, and more groups were contrasted through one-way ANOVA using GraphPad Prism 8.0. Asterisks (*) corresponded with *p* < 0.05 (*), *p* < 0.01 (**), *p* < 0.001 (***), and *p* < 0.0001 (****).

## Results

### EV-A71 infection increased the production and aggregation of Aβ_1–42_

**Fig. 1:**
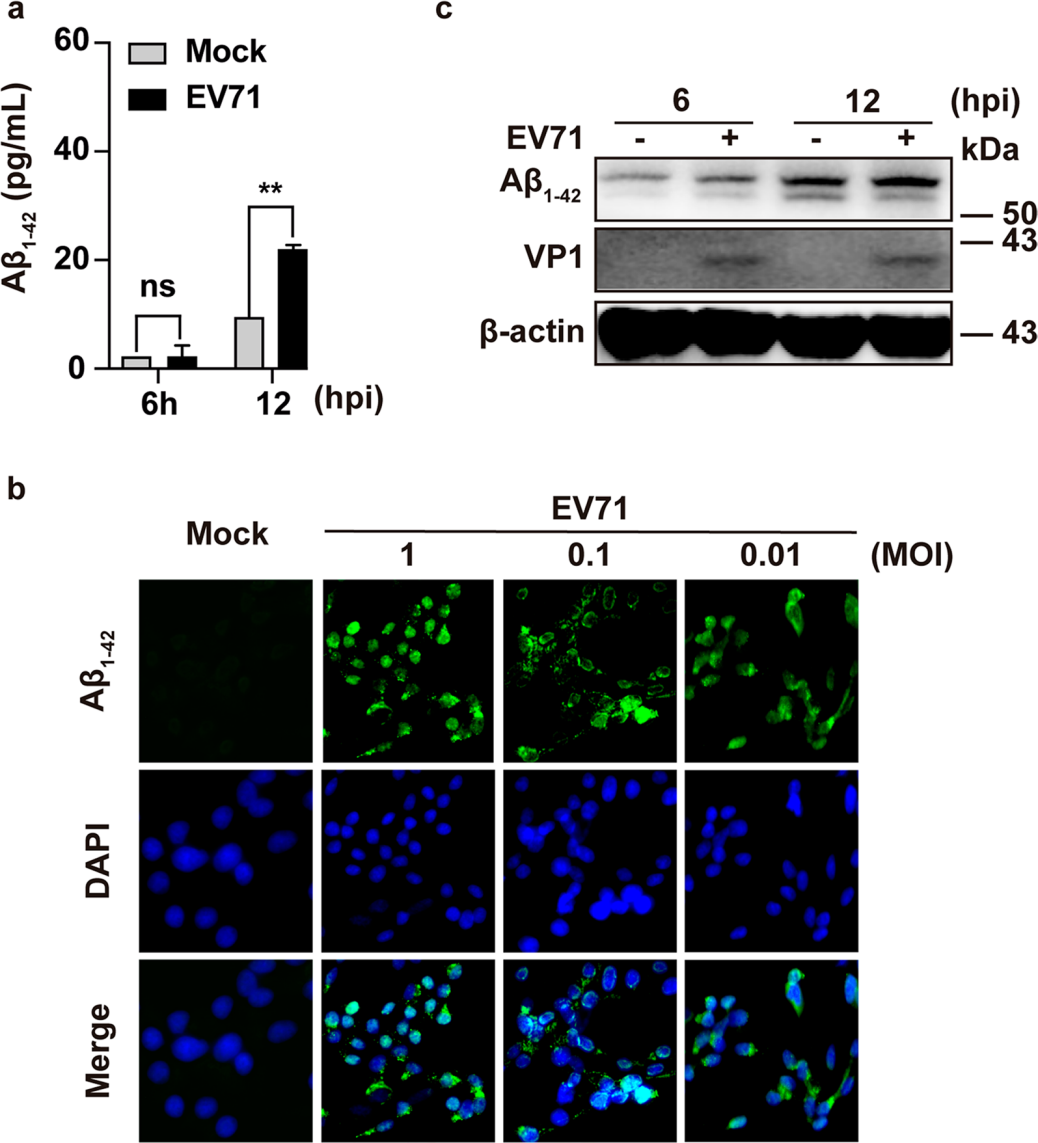
Aβ_1–42_ generation and aggregation were responsive for EV-A71 infection in SH-SY5Y cells. **a** Production of Aβ_1–42_ from the supernatant was quantified with an ELISA kit. SH-SY5Y cells were subjected to EV-A71 infection (MOI = 1) and collected at 6 or 12 h post-EV-A71 infection. **b** Production of intracellular Aβ_1–42_ was quantified by IFA assay. SH-SY5Y cells were subjected to EV-A71 infection at MOIs of 0.01, 0.1, and 1 for 8 h, respectively. IFA of Aβ_1–42_ protein was performed with an Alexa Fluor 488-conjugated antibody (green), and the nucleus was dyed with DAPI (blue). **c** Aβ_1–42_ high-molecular-weight oligomer accumulation in response to EV-A71 (MOI = 1) at 6 and 12 h after infection was detected by Western blot with anti-Aβ_1–42_ antibody (~ 70 kDa)

To determine whether Aβ expression was altered in response to EV-A71 infection, we used an ELISA assay to detect the secreted expression level of Aβ_1–42_ in the supernatant of EV-A71-infected SH-SY5Y cells (MOI = 1) and applied immunofluorescence assay to test the intracellular Aβ_1–42_ expression. As presented in Fig. 1a, compared with mock-infected control, the concentration of secreted Aβ_1–42_ was significantly higher at 12 h after EV-A71 infection. Moreover, Aβ_1–42_ was slightly induced after EV-A71 infection, and its expression increased with increased virus dose (Fig. 1b). The accumulation of Aβ_1–42_ high-molecular-weight oligomer aggregates (~ 70 kDa) was observed by WB assay at 6 and 12 h after EV-A71 infection (MOI = 1) (Fig. 1c). A similar phenomenon has been found in HSV-infected cells [20]. These results suggested that EV-A71 infection can induce Aβ_1–42_ production and aggregation accumulation.

### Aβ_1–42_ inhibited EV-A71 replication in vitro

**Fig. 2:**
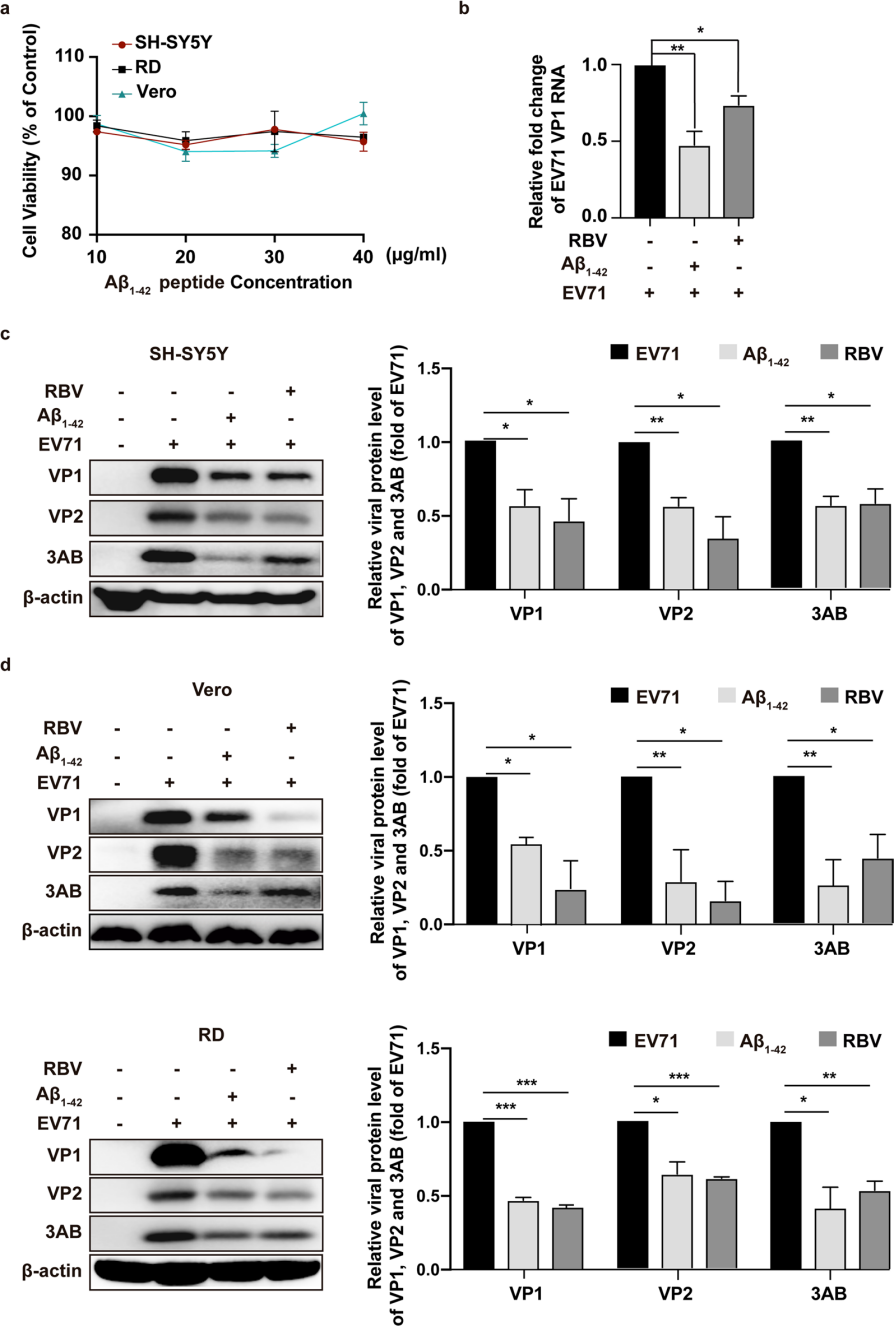
Cytotoxicity and anti-EV-A71 activity of Aβ_1–42_ in vitro. **a** Cytotoxicity of Aβ_1–42_ to multiple cell lines were determined by CCK assay at 48 h post-peptide treatment. **b** and **c** SH-SY5Y cells were infected with EV-A71 (MOI = 1) and followed by treatment with Aβ_1–42_ peptides or RBV (20 µg/mL) for 24 h. The concentrations of EV-A71 VP1 RNA and protein were assayed by qRT-PCR and WB, respectively. **d** Aβ_1–42_ peptides or RBV were added to EV-A71-infected Vero (MOI = 0.1) and RD cells (MOI = 0.01) for 24 h. The content of EV-A71 VP1 protein was analyzed by WB. Software “Gel-Pro analyzer” was used to analysis of the optical density ratio of the bands

To determine whether Aβ oligomers exerted antiviral activity against EV-A71 infection, we examined the effects of Aβ_1–42_ oligomerized peptide on EV-A71 replication in neural cells and EV-A71 susceptible cell lines, Vero, and RD cells. To exclude possible cytotoxicity-mediated antiviral effect, we initially determined the cytotoxicity of Aβ_1–42_ in SH-SY5Y, Vero, and RD cells through CCK assay. Results showed that cell viability was approximately 93%–100% in the treatment of Aβ_1–42_ for 48 h at concentrations of 30 or 20 µg/mL (Fig. 2a). Accordingly, we applied Aβ_1–42_ at 20 µg/mL for subsequent antiviral assays. In SH-SY5Y cells, Aβ_1–42_ and ribavirin (RBV) significantly inhibited the RNA level of EV-A71 VP1 (Fig. 2b), as well as the protein levels of VP1, VP2, and nonstructure protein 3AB (Fig. 2c). The inhibitory percentages of Aβ_1-42_ on VP1, VP2 and 3AB proteins in SH-SY5Y cells were 42.81%, 43.64%, and 44.16%, respectively. Then, we moved to EV-A71 susceptible cell lines, and results showed that Aβ_1–42_ and RBV effectively inhibited the expression of VP1, VP2, and 3AB proteins in RD cells. The inhibitory percentages of Aβ_1-42_ on VP1, VP2 and 3AB proteins in RD cells were 53.50%, 36.36% and 58.57%, respectively. In Vero cells the inhibitory percentages were 45.81%, 71.42%, and 74.00%, respectively (Fig. 2d). This finding suggested that Aβ_1–42_ inhibited EV-A71 replication independent of cell-line specificity.

### Aβ_1–42_ targeted the early stage of the EV-A71 life cycle

**Fig. 3:**
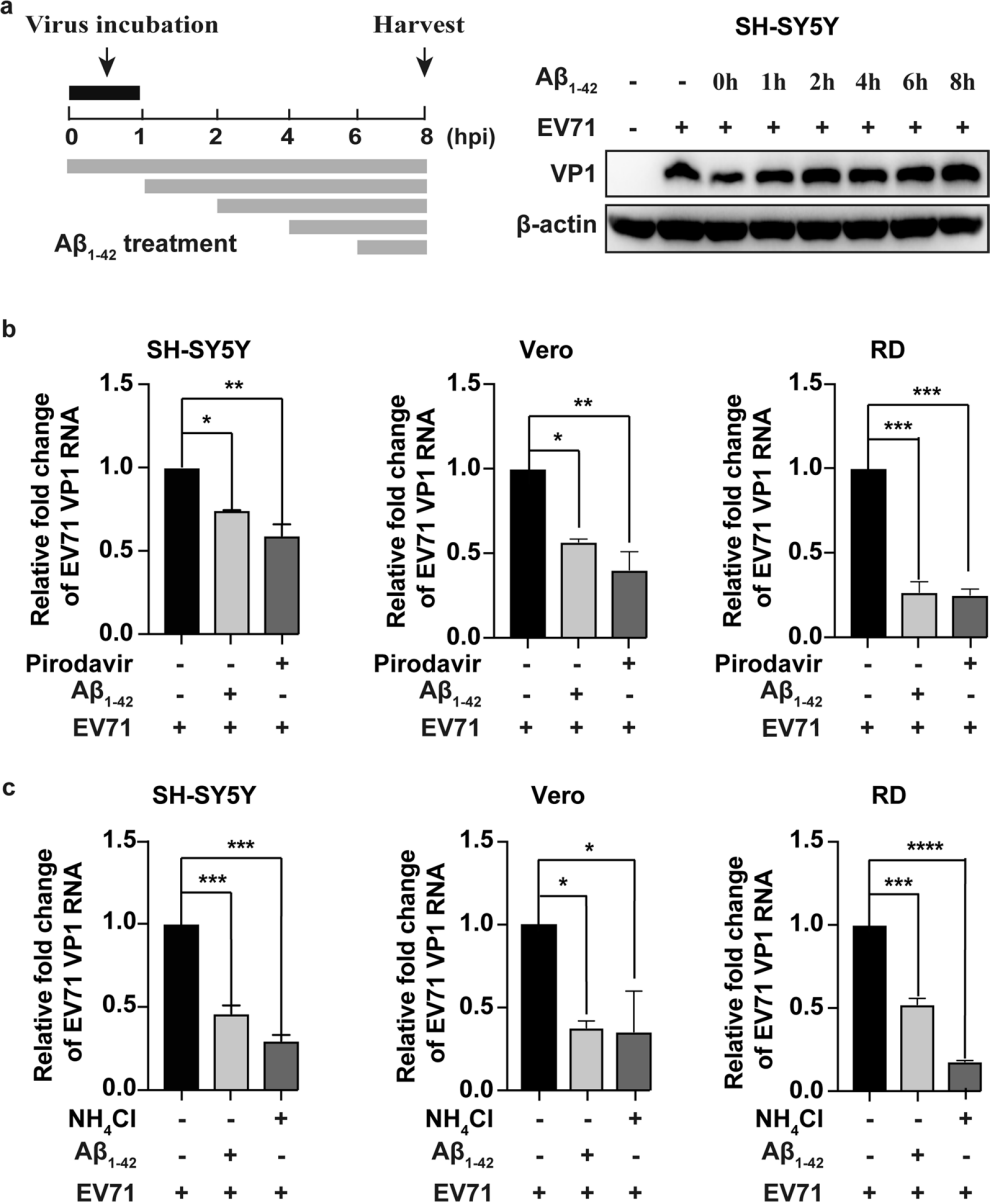
Aβ_1–42_ targeted the attachment and post-attachment phases of EV-A71 infection. **a** Time-of-addition experiment. SH-SY5Y cells were subjected to EV-A71 infection (MOI = 10) at 0 h time point. At 1 h point, cells were cleaned in PBS buffer and collected at 8 hpi. EV-A71 VP1 was determined by WB assay. The grey column indicates the period in which 30 µg/mL Aβ_1–42_ peptides were present. **b** EV-A71 virus (MOI = 2.5) was added to precooled cell plates simultaneously with Aβ_1–42_ (30 µg/mL) or pirodavir (40 µM) at 4 °C for 1 h. The amounts of cell-bound EV-A71 particles were measured by qRT-PCR. **c** Precooled cells that adhered to EV-A71 were incubated with Aβ_1–42_ (30 µg/mL) or NH_4_Cl (40 µM) at 37 °C for 60 min. The content of vp1 RNA was identified by qRT-PCR

In deciphering the EV-A71 life cycle stages targeted by Aβ_1–42_, we performed a time-of-addition experiment. We increased the inoculum to 10 MOI of EV-A71 to ensure a high infection efficiency to assess one-step growth [21]. Aβ_1–42_ was added at different time points either during or after EV-A71 infection and quantified the viral VP1 protein by WB assay 8 h after EV-A71 infection. As shown in Fig. 3a, when Aβ_1–42_ was added 1 h during or after EV-A71 infection, the antiviral effect was almost completely lost. This result indicated that Aβ_1–42_ inhibited EV-A71 infection at the early stage of the virus-replication cycle, possibly inhibiting virus entry.

To dissect which step of EV-A71 entry was affected by Aβ_1–42_, we evaluated the process of viral attachment in SH-SY5Y, Vero, and RD cells. In the viral-attachment assay, the content of VP1 RNA was measured by qRT-PCR, and the inhibition of Aβ_1–42_ on viral attachment was observed in three cell lines (MOI = 2.5). Pirodavir, the known capsid inhibitor of EV-A71, served as the control [22]. Compared with the untreated control, the viral VP1 RNA levels with Aβ_1–42_ or pirodavir treatments decreased in all three cell lines (Fig. 3b). In the penetration-inhibition assay, we further studied the effect of Aβ_1–42_ on the EV-A71 RNA level at 1 h post-virus attachment (MOI = 2.5). Figure 3c shows that consistent with NH_4_Cl treatment [23], Aβ_1–42_ efficiently downregulated the level of VP1 RNA after EV-A71 attachment in all three cell lines.

Taken together, these results demonstrated that Aβ_1–42_ primarily targeted EV-A71 attachment, internalization, and uncoating stage to inhibit virus replication.

### Aβ_1–42_ bound to EV-A71 VP1 protein

**Fig. 4:**
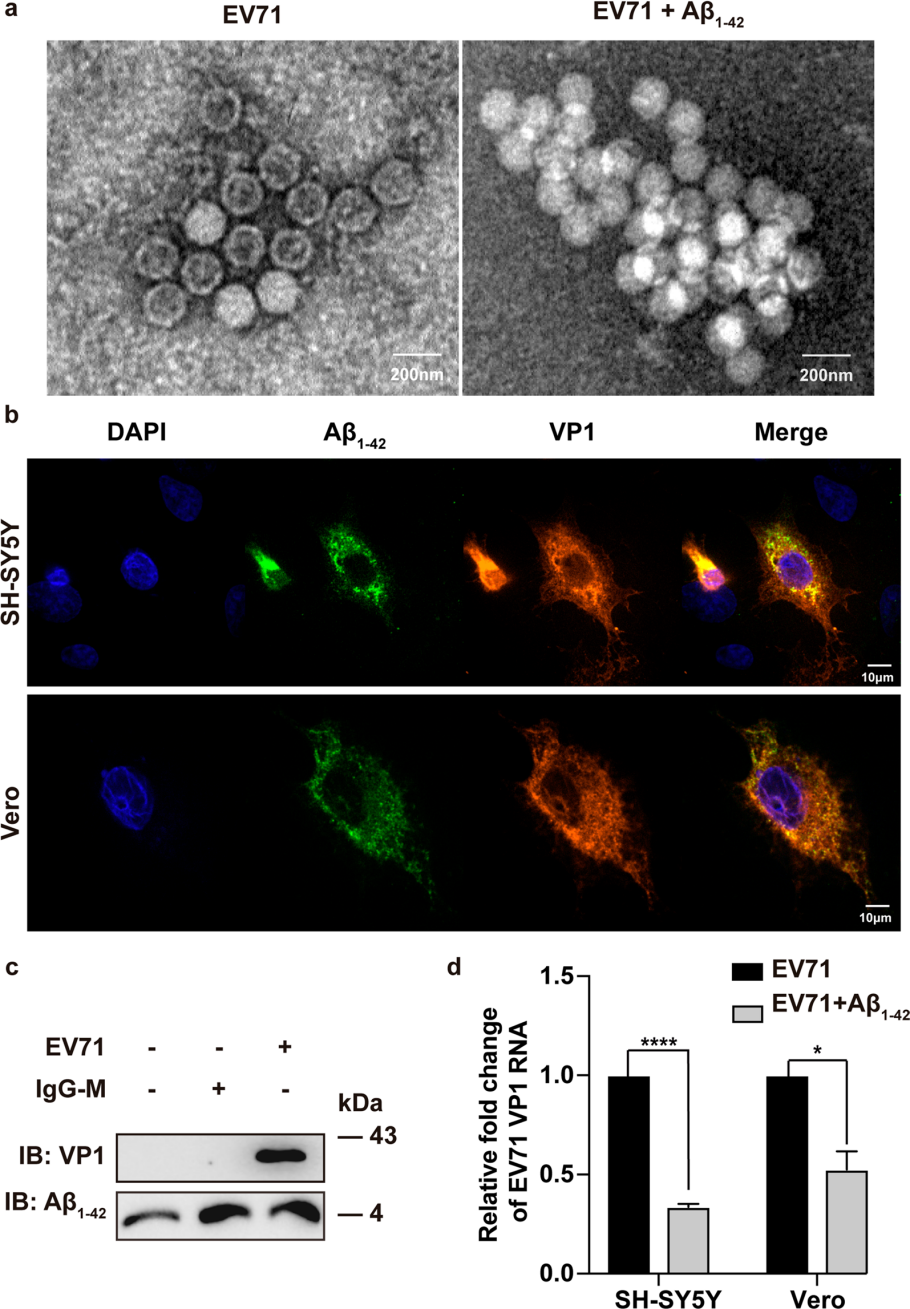
Aβ_1–42_ induced EV-A71 aggregation and directly bound to VP1. **a** Purified EV-A71 virus was incubated with Aβ_1–42_ (20 µg/mL) at 4 °C for 60 min, and EV-A71 aggregation was analyzed by TEM. **b** SH-SY5Y or Vero cells were subjected to EV-A71 infection (MOI = 2.5) for 8 h, and the co-localization between Aβ_1–42_ (detected with a 488-conjugated antibody against Aβ_1–42_, green) and EV-A71 VP1 (Alexafluor594-conjugated antibody against VP1, red) was identified by confocal assay. Images were captured at 100 × magnification with a PE UltraVIEW VOX. **c** Aβ_1–42_ (20 µg/mL) was mixed with activated protein A/G magnetic beads overnight at 4 °C. Subsequently, the mixture was cultivated with purified EV-A71 virus (MOI = 1) at 4 °C for h. After cleaning the beads to remove nonbound viruses, the magnetic beads were subjected to precipitation with a magnet holder and assayed by Western blot with VP1 antibody. **d** Pre-virus attachment inhibition assay of Aβ_1–42_ showed comparable inhibition capacity against EV-A71 infections. Virus (MOI = 2.5) with or without Aβ_1–42_ medium incubated at 4 °C for 1 h, followed by infecting precooled cells at 4 °C for another 1 h. The quantity of cell-bound EV-A71 virus was identified by qRT-PCR. Experiments were independently repeated three times

To further investigate the mechanism by which Aβ_1–42_ treatment induced EV- A71 entry blockage, we determined whether Aβ_1–42_ could cause the aggregation of EV-A71 and affect its entry. After incubating Aβ_1–42_ peptides with EV-A71 for 1 h, the aggregation of EV-A71 particles was observed by TEM assay (Fig. 4a). Moreover, the colocalization of VP1 and Aβ_1–42_ was observed in SH-SY5Y and Vero cells (Fig. 4b). We further confirmed that Aβ_1–42_ interacted with EV-A71 capsid protein VP1 by pull-down assay (Fig. 4c). Co-incubation of EV-A71 with Aβ_1–42_ at 4 °C for 1 h also significantly reduced the infectivity of EV-A71 (Fig. 4d). Consequently, we revealed that Aβ_1-42_ promoted the aggregation of EV-A71 virus particles and bound to the capsid protein VP1, which partially caused Aβ_1–42_ to prevent viruses from infecting cells.

### Aβ_1–42_ bound to the EV-A71 receptor SCARB2

**Fig. 5:**
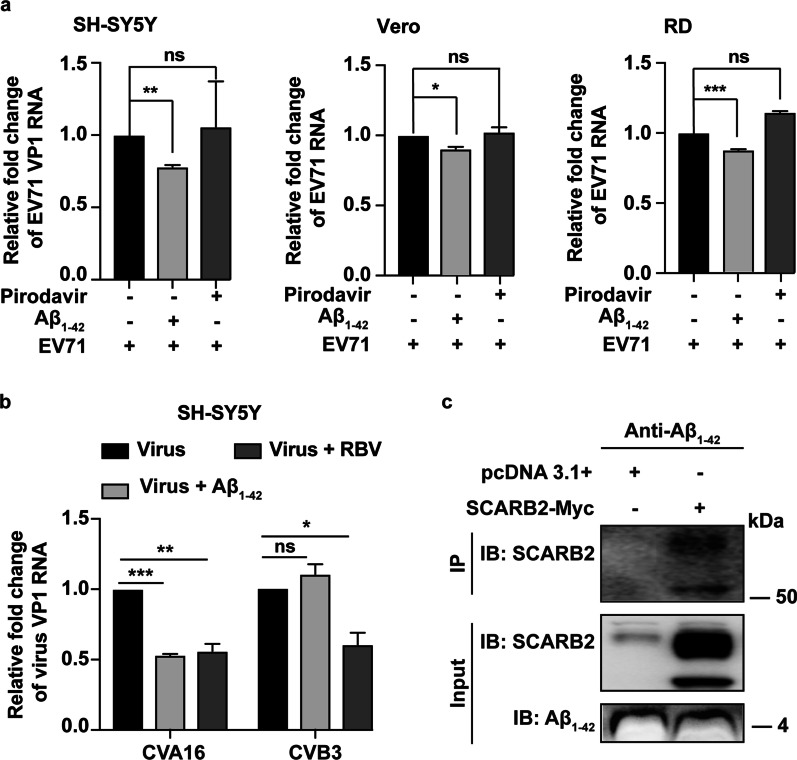
Aβ_1–42_ is directly bonded to SCARB2. **a** SH-SY5Y, Vero, and RD cells were pretreated with Aβ_1–42_ (20 µg/mL) or pirodavir (40 µM) at 4 °C for 60 min, followed by EV-A71 infection (MOI = 2.5) at 4 °C for another 1 h. The overall RNA was harvested, and EV-A71 VP1 RNA variation was evaluated by qRT-PCR. **b** SH-SY5Y cells were subjected to CVA16 or CVB3 infection (MOI = 1) and afterward subjected to Aβ_1–42_ peptide or RBV treatment (20 µg/mL) for 24 h. The virus VP1 RNA level was analyzed by qRT-PCR. **c** Vero cells were subjected to SCARB2-myc or pcDNA 3.1 + plasmid transfection for 24 h and lysed with protein lysate containing phosphatase and protease inhibitor. Subsequently, the lysis buffer supernatant was mixed with Aβ_1–42_ immobilized magnetic beads at 4 °C for 2 h. The bound beads were suspended with a 1 × sample loading buffer and boiled for 10 min. The binding of SCARB2 was detected by WB assay with anti-SCARB2 antibody

The carbohydrate-binding function of Aβ can inhibit microbial infections by targeting glycoproteins on the cell wall of microbes or enveloped viruses [24]. Accordingly, we performed a series of methods to determine whether the antiviral activity of Aβ_1–42_ was related to SCARB2, which was an attachment and uncoating glycoproteins for EV-A71 infection. We initially performed a pre-Aβ_1–42_ peptide-attachment assay to determine whether Aβ_1–42_ pre-interacted with SCARB2 on the cell surface. We pretreated the cells with Aβ_1–42_ or pirodavir for 1 h and then infected them with EV-A71 (MOI = 2.5). The adsorption of EV-A71 on cellular surfaces was detected by qRT-PCR. The outcomes unveiled that pretreatment with Aβ_1–42_ significantly suppressed the adsorption of EV-A71 in all three cell lines, whereas pretreatment with pirodavir did not affect EV-A71 adsorption (Fig. 5a). Furthermore, consistent with inhibiting EV-A71, Aβ_1–42_ also exhibited the same effect on CVA16 but not on CVB3 (Fig. 5b) possibly because SCARB2 was only a receptor of CVA16 rather than CVB3 [11–13]. We further performed a pull-down experiment to demonstrate whether an interaction existed between Aβ_1–42_ and SCARB2. Aβ_1–42_ immobilized magnetic beads were used to pull down SCARB2 protein overexpressed in Vero cells. Results showed that Aβ_1–42_ could specifically bind to SCARB2 protein (Fig. 5c). These results suggested that Aβ_1–42_ might inhibit EV-A71 by interacting with SCARB2 to block virus entry.

## Discussion

Enterovirus A types, including EV-A71 and CVA16, are primary pathogens related to HFMD and encephalitis, which are typical neural complications from HFMD infection. They are accompanied by complications such as central nervous system (CNS) damage, particularly in East and Southeast Asia [15, 25, 26]. Aβ has long been recognized as a pathogenetic factor for AD. However, the antimicrobial function of Aβ has been gradually revealed recently. Here, we demonstrated for the first time that Aβ_1–42_ exhibited antiviral activity against EV-A71 and CVA16 infections. The anti-EV-A71 effect of Aβ presented in SH-SY5Y cells, and in Vero and RD cells indicates that Aβ_1–42_ inhibited EV-A71 replication independent of cell-line specificity. Mechanistically, we revealed that Aβ_1–42_ interfered with the attachment and uncoating stage of EV-A71 infection. The effect of Aβ_1–42_ on suppressing viral post-attachment was more effective than the attachment in SH-SY5Y cells, whereas an equal inhibition effect was observed in Vero and RD cells. The reason for this apparent difference in nerve cells remains elusive. Aβ exhibited a direct ability to bind microbial surface carbohydrates, primarily mediating its antimicrobe infection capability. The carbohydrate binding of Aβ promoted self-fibrillization, leading to the aggregation of enveloped viruses such as HSV-1, HHV6, and IAV, thereby reducing viral infectivity [4, 6, 27, 28]. However, we discovered that Aβ_1–42_ also induced a slight aggregation of non-envelope virus EV-A71 after co-incubating for 1 h and bound to virus capsid protein VP1 performed by pull-down assay. Meanwhile, either the binding to VP1 or the aggregation of EV-A71 may lead to the impairment of virus attachment (Fig. 4d). Bourgade et al*.* suggested that the carbohydrate-binding of Aβ may not be the only pathway to target herpesviruses. Previous research has assumed that Aβ, which has high sequence homology to the HSV-1-gB membrane-proximal region, targets HSV-1 by competing for binding gB's fusion loops, thereby preventing the HSV-1 fusion cell process [29]. Based on VP1 of EV-A71, which is not a glycoprotein, the binding between VP1 and Aβ may exist in other pathways. Accordingly, we speculated that a noncarbohydrate binding region of Aβ_1–42_ may mediate the targeting of EV-A71 capsid protein VP1.

In HSV-1 and IAV, the present research showed that Aβ inhibited virus replication outside the cells but not when peptides were added after virus infection. In contrast to previous reports, we discovered that the antiviral action of Aβ could effectually occur inside the target cells (Figs. 2 and 3c). Unlike enveloped viruses, the cellular entry of non-enveloped viruses primarily relies on the binding to cell surface virus receptors. SCARB2, a membrane glycoprotein, mediates viral entry into the cell and is a common receptor for all enterovirus A virus. Consistent with the glycoprotein-binding function, Aβ could interact with SCARB2 and inhibit EV-A71 attachment and entry (Fig. 5). We further found that Aβ_1–42_ inhibited CVA16 rather than CVB3 replication (Fig. 5b), possibly because SCARB2 was not a receptor for CVB3. Hsu et al*.* revealed that Aβ could bind to the spike protein S1 sub-unit (S1) of the SARS-CoV-2 and the viral receptor ACE2, enhancing the interaction of the S1 protein with ACE2 and increasing the virus entry along with intracellular IL-6 production [30]. Figure 5a indicates that pretreating with Aβ_1–42_ peptides in various cell lines led to evident reductions in EV-A71 RNA levels. This finding suggested that Aβ exerted a competitive combination with SCARB2 instead of strengthening the binding of EV-A71 to SCARB2. Further investigation is required to confirm the interference of Aβ1–42 on the binding of the virus to SCARB2. Recent research has suggested that the VP1 GH and VP2 EF loops of EV-A71 interact with SCARB2 and further facilitate the low-pH uncoating of the virus in endosomes/lysosomes [31]. Our results showed that Aβ_1–42_ bound to VP1 protein but not VP2 (Additional file 1: Fig. S1), indicating that the viral VP1 GH loop may play a key role in mediating the interaction of VP1 and Aβ_1–42_. Our results further showed that Aβ_1–42_ interacted with SCARB2 or VP1. However, further studies are needed to confirm whether these two interactions were competitive or synergistic.

Toczylowski K et al. measured Aβ1–42, a traditional AD biomarker, in children with enteroviral meningitis caused by echovirus. The CSF concentration of Aβ1–42 was decreased when compared with the control group without CNS infection. In contrast, other biomarker concentrations are unchanged [32]. Thus, an enteroviral infection may lead to a specific impact on Aβ1–42 production. Our results showed that Aβ_1–42_ generation and accumulation were induced at 12 h after EV-A71 infection in SH-SY5Y cell lines (Fig. 1). The findings of Toczylowski et al*.*, together with ours, reveal the distinct effect of various enteroviruses infection on Aβ_1–42_ production. Nevertheless, whether Aβ production and further aggregation is a protective immune response against the invasion of foreign pathogens such as EV-A71 infection requires further exploration.

Over the past few years, several infection factors have been proposed to be associated with AD development [33]. Most past research has focused on the relation between Aβ_1–42_ and HSV-1. Little is known about enteroviruses that exert a similar inflammatory influence on CNS due to HSV infection [24, 33]. Thus, more detailed studies on the specific binding of the amino acid region of Aβ_1–42_ to capsid VP1 protein or virus receptor SCARB2 should be performed. Apart from SCARB2, PSGL-1 is also a typical glycoprotein receptor of EV-A71. However, PSGL-1 is expressed by macrophages in the intestinal mucosa and lymph-node dendritic cells, and it plays a key role in trafficking inflammation stimulated by infection [34]. We did not use PSGL-1-expression cell lines in this research and will explore it in a follow-up work if needed. The previous discoveries of Bourgade et al*.* have revealed that Aβ_1–42_ can not inhibit non-enveloped human adenovirus replication in vitro. Our findings unveiled that Aβ1–42 can sufficiently inhibit non-enveloped EV-A71 effectively, thereby extending the potential function of Aβ in other non-enveloped viruses linked to infectious neurological diseases.

## Conclusion

We demonstrated for the first time that Aβ_1–42_ exhibited antiviral activity against EV-A71 infection. We primarily revealed that Aβ_1–42_ interfered with the attachment and uncoating stage of EV-A71 infection. Given that non-polio enteroviruses including EV-A71 are known to display strong neurotropism, the role of Aβ in EV-A71 infection is worth investigating.

## Supplementary Information

**Additional file 1: Fig. S1.** Aβ_1–42_ directly interacted with VP1 but not VP2. Vero cells were transfected with HA-VP1, HA-VP2, or pcDNA 3.1 + plasmid for 24 h and lysed with protein lysate containing phosphatase and protease inhibitor. Then, the lysis buffer supernatant was mixed with Aβ_1–42_ immobilized magnetic beads at 4 °C for 2 h. The bound beads were suspended with a 1 × sample loading buffer and boiled for 10 min. The binding of VP1 or VP2 was detected by WB assay with anti-HA antibody

## Data Availability

The datasets supporting the conclusions of this article are included within the article.

## Abbreviations

Aβ: β-Amyloid protein
AD: Alzheimer’s disease
AMP: Antimicrobial peptide
EV-A71: Enterovirus A 71
CCK: Cell Counting Kit
EM: Enteroviral meningitis
qRT-PCR: Real-time reverse transcription-PCR
SCARB2: Scavenger receptor class B member 2
TEM: Electron Microscope
