# Supplementary figures and images for: Effects and mechanism of Aβ1−42 on EV-A71 replication
Source: Virol J. 2022 Sep 20;19:151. doi: 10.1186/s12985-022-01882-3 (PMC9485788; doi:10.1186/s12985-022-01882-3)

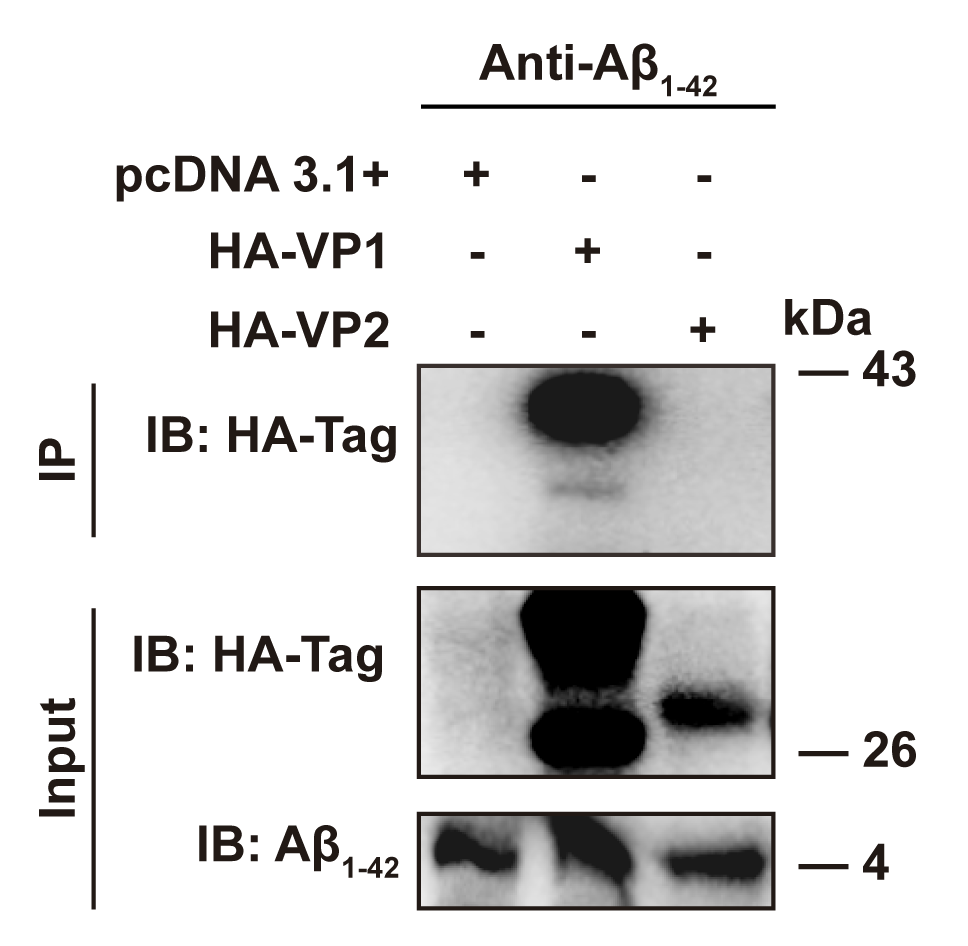

Supplement: Supplementary file 1 — Additional file 1: Fig. S1. Aβ1–42 directly interacted with VP1 but not VP2. Vero cells were transfected with HA-VP1, HA-VP2, or pcDNA 3.1 + plasmid for 24 h and lysed with protein lysate containing phosphatase and protease inhibitor. Then, the lysis buffer supernatant was mixed with Aβ1–42 immobilized magnetic beads at 4 °C for 2 h. The bound beads were suspended with a 1 × sample loading buffer and boiled for 10 min. The binding of VP1 or VP2 was detected by WB assay with anti-HA antibody [file 12985_2022_1882_MOESM1_ESM.tif]
